# Synthesis of new, highly luminescent bis(2,2’-bithiophen-5-yl) substituted 1,3,4-oxadiazole, 1,3,4-thiadiazole and 1,2,4-triazole

**DOI:** 10.3762/bjoc.10.165

**Published:** 2014-07-14

**Authors:** Anastasia S Kostyuchenko, Vyacheslav L.Yurpalov, Aleksandra Kurowska, Wojciech Domagala, Adam Pron, Alexander S Fisyuk

**Affiliations:** 1 Department of Organic Chemistry, Omsk F. M. Dostoevsky State University, 55a Mira Ave, 644077 Omsk, Russia; 2 Laboratory of New Organic Materials, Omsk State Technical University, Mira Ave, 11, Omsk 644050, Russia; 3 Department of Physical Chemistry and Technology of Polymers, Silesian University of Technology, Marcina Strzody 9, 44-100 Gliwice, Poland; 4 Faculty of Chemistry Warsaw University of Technology, Noakowskiego 3, 00-664 Warszawa, Poland

**Keywords:** bithiophene, donor–acceptor, luminescence, 1,3,4-oxadiazole, 1,3,4-thiadiazole, 4*H*-1,2,4-triazole

## Abstract

A new synthetic approach towards the preparation of functionalised, soluble, donor–acceptor (DA) alkylbithiophene derivatives of oxadiazole, thiadiazole and triazole is reported. Taking advantage of the Fiesselmann reaction, reactive bithiophene synthons having alkyl or alkoxy substituents at designated positions are prepared. Following a synthetic strategy, featuring the bottom-up approach, sequential structural elements are built, starting from a simple thiophene compound, until the target molecule is obtained, all in good yield. Supplementing the well established methods of oxadiazole and thiadiazole synthesis, efficient ring closure reaction affording a 4*H*-1,2,4-triazole unit is presented. All target ambipolar compounds display strong photoluminescence with measured quantum yields up to 0.59. Modification of the demonstrated synthetic routes may be exploited for the preparation of longer, specifically functionalised oligothiophenes, coupled to other heteroaromatic cores.

## Introduction

In the past two decades oligo- and polythiophenes gained a significant research interest due to their wide application as organic semiconductors in light emitting diodes (OLED’s), field effect transistors (FETs), electrochemical and chemical sensors and other organic electronic devices [1–5]. This family of organic semiconductors is especially interesting because they can be rendered solution processable, yielding strictly controlled supramolecular organisation, provided that appropriate deposition techniques are applied, such as zone casting [6–8], mechanical rubbing or directional epitaxial crystallisation [9]. For many applications, however, the HOMO and LUMO levels of oligo- and polythiophenes and by consequence their electron affinity (EA) and ionization potential (IP) are not appropriate [2]. Since these two parameters determine the redox, spectroscopic, electronic and optoelectronic properties of oligothiophenes, their tuning is of crucial importance in any design of new organic semiconductors. One of the possible ways of the preparation of low molecular mass semiconductors, showing higher than oligothiophene IP values, is to synthesize molecules in which a central electron accepting group separates two bi-, ter- or quaterthiophene units. Such compounds containing thiadiazole [10–15], oxadiazole [14–17] or tetrazine units [13,18–20] have been reported. Moreover, luminescence properties of these derivatives are superior to those of the corresponding penta-ring oligothiophenes. For example, the central heterocycle ring replacement in substituted quinquethiophene by 1,3,4-oxadiazole leads to a substantial increase of the fluorescence quantum yield and a hypsochromic shift of the emission band [14], both features being technologically advantageous. They are, however, difficult to solution process due to strong intermolecular interactions, associated with their extended conjugation and frequently have to be rendered soluble by introducing long alkyl- or alkoxy-type side substituents. This functionalisation opens up an additional route to modifying their redox, electronic and optical properties by using side substituents with different electron accepting/electron donating properties [11].

In this article, we are expanding our prior syntheses of alkylbithiophene substituted thiadiazoles [11] to evaluate the effect of other heteroaromatic rings at the central position on the resulting properties of these five-ring compounds. DA compounds with a 1,3,4-oxadiazole or 1,2,4-triazole central ring are reported herein. The synthesized derivatives are very interesting new semiconductors of possible use as electroluminophores since preliminary studies showed that they could be used as active components of host–guests organic light emitting diodes [21].

## Results and Discussion

### Synthetic toolbox

**Scheme 1:**
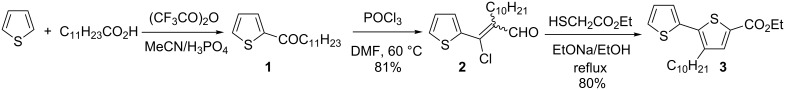
Synthesis of ethyl 3-decyl-2,2'-bithiophene-5-carboxylate (**3**).

There are several approaches to the synthesis of 2,5-bis(2,2 '-bithiophen-5-yl)-1,3,4-oxadiazoles and 1,3,4-thiadiazoles. Transition metal-catalysed cross-coupling reactions, such as Stille, Suzuki etc. are typically used for the formation of thiophene–thiophene or azole–thiophene bonds [14,16–17]. However, the best results (higher yields, more facile purification, shorter reaction times) are obtained by the formation of the azole ring via appropriately functionalized bithiophenes [14,22]. Recently, we have developed a new method for the preparation of 3-alkyl(aryl) substituted esters of 2,2'-bithiophene-5-carboxylic acids, which is based on a modification of the Fiesselmann reaction [11,23]. Ethyl 3-decyl-2,2'-bithiophene-5-carboxylate (**3**) was obtained by this method in 80–85% yields at each step [24] (Scheme 1).

**Scheme 2:**
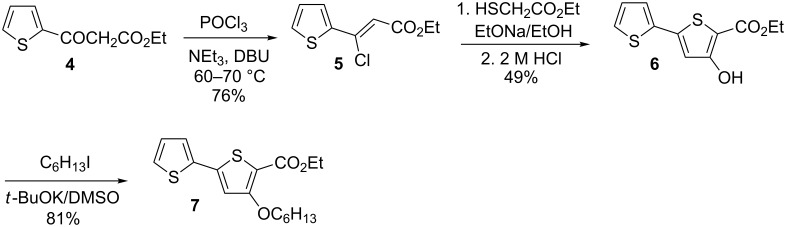
Synthesis of ethyl 4-(hexyloxy)-2,2'-bithiophene-5-carboxylate (**7**).

Ethyl 3-oxo-3-(2-thienyl)propanoate (**4**), obtained by a known method [25], was used as starting compound for the synthesis of ethyl 4-hydroxy-2,2'-bithiophene-5-carboxylate (**6**). Reaction of **4** with POCl_3_ led to the formation of ethyl 3-chloro-3-(2-thienyl)acrylate (**5**) which was then converted to ethyl 4-hydroxy-2,2'-bithiophene-5-carboxylate (**6**) by reacting with ethyl mercaptoacetate in a basic medium. The yields of **5** and **6** were 76% and 49%, respectively. Ethyl 4-(hexyloxy)-2,2'-bithiophene-5-carboxylate (**7**) was obtained by the alkylation of **6** with hexyl iodide in the presence of potassium *tert*-butoxide in 81% yield (Scheme 2). Compounds **5**–**7** were chromatographically purified using a silica gel column.

**Scheme 3:**
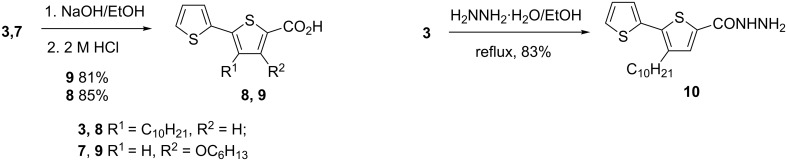
Hydrolysis and hydrazinolysis of esters **8**–**10**.

Esters **3** and **7** were converted to the corresponding carboxylic acids **8** and **9** by heating in an alcoholic solution of sodium hydroxide. The hydrolysis of **7** to the corresponding acid runs more difficult than in the case of **3** and requires longer reaction times due to lower electrophilicity and a bigger steric hindrance at the carbethoxy group. The hydrazide derivative **10** was obtained by refluxing **3** with hydrazine monohydrate in alcohol. Hydrazinolysis of **7** under these conditions runs slow and leads to a mixture of products (Scheme 3).

**Scheme 4:**
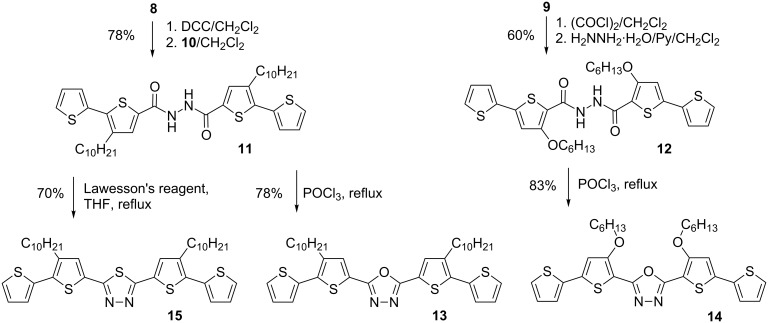
Synthesis of 2,5-bis(2,2'-bithiophen-5-yl)-1,3,4-oxadiazoles **13** and **14** and 1,3,4-thiadiazole **15**.

Diacylhydrazine **11** was prepared by the reaction of carboxylic acid **8** and hydrazide **10** in the presence of dicyclohexylcarbodiimide (DCC) in 78% yield. Compound **12** was obtained in 60% yield by reacting hydrazine monohydrate and the carboxylic acid chloride which was prepared in situ from **9** with oxalyl chloride (Scheme 4).

**Scheme 5:**
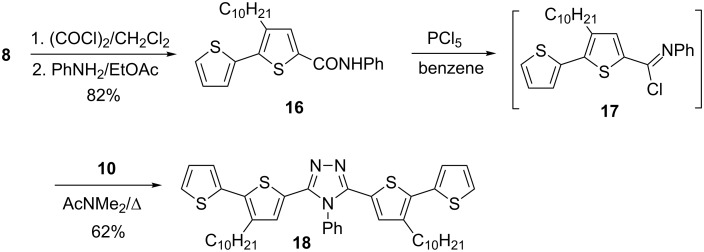
Synthesis of 3,5-bis(3-decyl-2,2'-bithiophen-5-yl)-4-phenyl-4*H*-1,2,4-triazole (**18**).

It is known that 1,3,4-oxadiazoles [26], 1,3,4-thiadiazole [27] and 1,2,4-triazoles [28–30] can be obtained from diacylhydrazines. Thus, **11** and **12** were converted to 2,5-bis(3-decyl-2,2'-bithiophen-5-yl)-1,3,4-oxadiazole (**13**) and 2,5-bis[4-(hexyloxy)-2,2'-bithiophen-5-yl]-1,3,4-oxadiazole (**14**) by the reaction with phosphorus oxychloride, in 78 and 83% yields, respectively. The reaction of **11** with a Lawesson’s reagent led to the formation of 2,5-bis(3-decyl-2,2'-bithiophen-5-yl)-1,3,4-tiadiazole (**15**, 70% yield) (Scheme 4). To the contrary, 3,5-bis(3-decyl-2,2'-bithiophen-5-yl)-4-phenyl-4*H*-1,2,4-triazole (**18**) could not be obtained by reacting **11** with O=P(NHPh)_3_ [30]. The major reaction product was the corresponding oxadiazole derivative **13**. An attempt to replace the oxygen atom by the nitrogen one upon refluxing **13** with aniline [31–32] was also unsuccessful. For these reasons, a different approach [33] had to be used for the synthesis of 3,5-bis(3-decyl-2,2'-bithiophen-5-yl)-4-phenyl-4*H*-1,2,4-triazole (**18**). Anilide **16** was used as a building block for the synthesis of **18**. It was prepared from **8** by a consecutive reaction with oxalyl chloride and then aniline. In the next step **16** was converted to 3-decyl-*N*-phenyl-2,2'-bithiophene-5-carboximidoyl chloride (**17**) by the reaction with phosphorus pentachloride, and then, without additional purification, used at once in the reaction with acyl hydrazide **10**. As a result, 3,5-bis(3-decyl-2,2'-bithiophen-5-yl)-4-phenyl-4*H*-1,2,4-triazole (**18**) was obtained in 62% yield (Scheme 5).

All intermediate and final products were identified by elemental analysis, NMR and IR spectroscopy (see Supporting Information File 1).

### Spectroscopic and luminescent properties of the synthesized compounds

**Figure 1:**
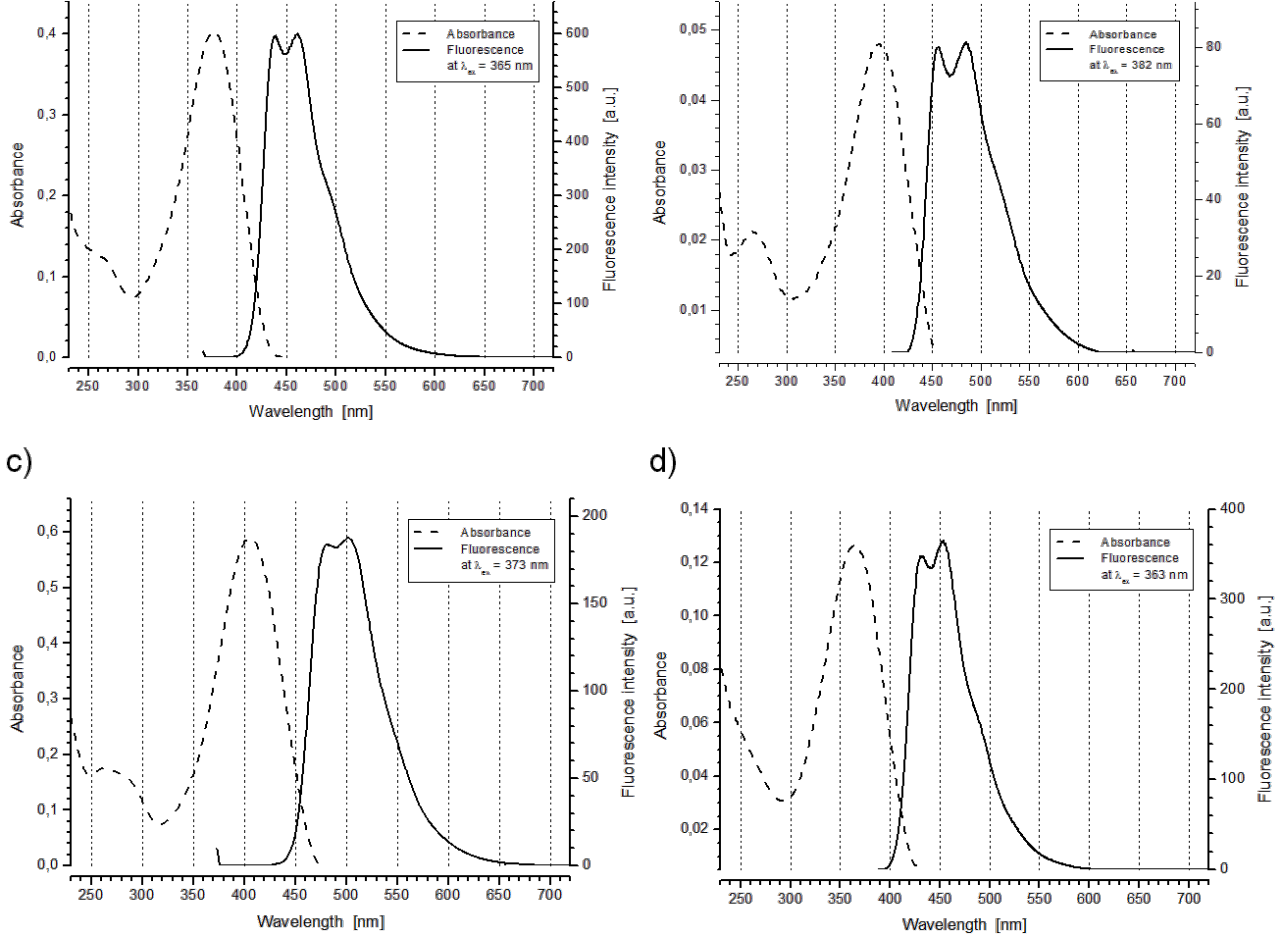
UV–vis absorption and fluorescence spectra of diluted solutions of a) **13**, b) **14**, c) **15**, and d) **18** in dichloromethane. Optimum fluorescence excitation wavelengths (given in the legends) have been selected based on fluorescence emission profiles.

The target molecules **13**, **14**, **15** and **18** feature light absorption in the near ultraviolet, tailing into high energy visible spectral range, giving them yellow to orange-tinted colouration in the solid state. When dissolved, they produce faint yellow coloured solutions exhibiting clear fluorescence properties, perceivable as a blue to bluish-green hue when exposed to direct sunlight. The absorption and maximum emission fluorescence spectra of their solutions in dichloromethane are collected in Figure 1, while their representative spectral parameters have been compiled in Table 1.

**Table 1:** Absorption spectral parameters of investigated donor–acceptor–donor molecules.

Compound	λ_max_ [nm]	λ_onset_ [nm]^a^	HOMO–LUMO transition [eV]^a^

**13**	376	432	2.87
**14**	395	455	2.72
**15**	406	469	2.64
**18**	365	421	2.94

^a^Pertaining to onset of the π–π* absorption peak.

All four compounds display an intense absorption band at the visible range edge, ascribable, on account of its energy, to a π–π^*^ transition. The wavelength maximum of this band clearly changes with the azole unit, as well as with the thiophene solubilising substituent, from 365 nm for **18**, 376 nm for **13**, 395 nm for **14** up to 406 nm for **15**. The difference of 19 nm between the two 1,3,4-oxadiazole derivatives **13** and **14**, is caused by the electron-donating alkoxy chain, increasing the molecule HOMO level resulting in narrowing of the frontier orbital gap. For the three targets with invariant bithiophene units (**18**, **13** and **15**), the smallest absorption band energy is observerd for **15**, i.e., the compound with the thiadiazole central unit. Such a decrease of the absorption band energy is typical of all sulfur containing five membered heterocycles [14–15,34].

Upon excitation with the UV light, all investigated compounds feature strong photoluminescence, emitting blue and green light (Table 2). Their emission energy maxima arrange themselves in the very same order as did the absorption energy maxima, i.e.: **18** > **13** > **14** > **15**. Compared to the featureless absorption spectra, structured fluorescence peaks are observed with two clearly discernible components and a third one concealed in the low energy tail (Figure 1). This fine structure appears to be a manifestation of enhanced vibronic coupling, which clearly suggests rigidification of the excited state molecular geometry of the compounds. This can be anticipated, considering the quinoid bond configuration of the excited state populated LUMO energy level of each molecule, in which the order of bonds connecting the heteroaromatic units increases. The energy dissipated upon geometric relaxation of the excited molecule following the vertical HOMO–LUMO transition, prior to the return transition from S_1_ to S_0_ state, is manifested as a Stokes shift. Since 0−0 transitions are rarely observed in room temperature solution spectra, it is acceptable to use Δ = λ_em_ − λ_max_ (see Table 1 and Table 2) as the Stokes shift magnitude index [15]. Moderate values of Stokes shifts are observed for the investigated compounds indicating that the geometric reconstruction takes place upon their optical excitation, the dominant reason being the bond order switching accompanying the benzenoid–quinoid transition. Small differences in the magnitude of this shift are observed arranging in a sequence **15** > **18** > **13** ≈ **14**, and scaling well with the atomic radii of the azole completive heteroatom. Moderate to high fluorescence quantum efficiency yields are also observed, ranging from 0.59 for **18** to 0.21 for **15**. Here, a clear heteroatom effect is observed, while the influence of the thiophene substituent seems negligible. The observed trend of decreasing quantum yield with increasing atomic number of the heteroatom of the azole unit is a clear manifestation of the heavy atom effect [35]. The proximity and availability of higher energy 3d levels in sulfur facilitates radiationless relaxation modes of the excited electron. Enhanced spin–orbit coupling opens up intersystem crossing channels as well, making the thermal decay pathway accessible. The hindering of these decay modes in the oxadiazole derivatives **13** and **14** doubles their quantum yield. For the triazole derivative **18** the quantum yield is even nearly triple that of **15**, but this enhancement should also be traced to the impaired conjugation of the triazole moiety with the bithiophene arms.

**Table 2:** Fluorescence spectral parameters of investigated donor–acceptor–donor molecules.

Compound	λ_ex_ [nm]^a^	λ_em_ [nm]^b^		Δ [nm]^c^	Ф_f_^d^

**13**	365	438	461	62	0.42
**14**	382	455	484	60	0.45
**15**	373	481	502	75	0.21
**18**	363	431	454	66	0.59

^a^Excitation; ^b^emission; ^c^minimum Stokes shift; ^d^quantum yield determined relative to 9,10-diphenylanthracene standard.

**Figure 2:**
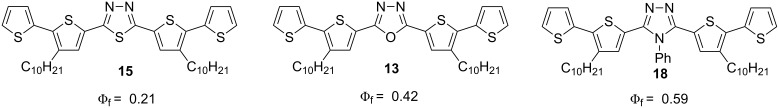
Photoluminescence quantum yields measured for **13**, **15** and **18** together with their formulae.

Comparing the obtained results with those reported for similar donor–acceptor–donor compounds, we can see that both the number of donor groups and the chemical nature of the central electron accepting ring exert strong influence on their fluorescence performance. With an increasing number of thiophene units higher quantum yield materials are obtained. For example, the quantum yield of 2,5-bis(3-methylthien-2-yl)-1,3,4-oxadiazole is 0.13 as compared to 0.46 reported for 2,5-bis[5-(3-octylthien-2-yl)-3-methylthien-2-yl]-1,3,4-oxadiazole [16]. Similarly, the position of the substitution with solubilising groups and the length of the side chains lead to a significant change of the quantum yield [11]. The effect of the central ring nature on the optical properties of **13**–**15** and **18** is clearly observed. In Figure 2 photoluminescence quantum yields are indicated together with the formulae of the corresponding compounds. In the case of **18** (4-phenyl-4*H*-1,2,4-triazole ring) the photoluminescence quantum yield is almost three times higher than that measured for **15** (1,3,4-thiadiazole ring).

The obtained results clearly demonstrate the need for careful and conscious molecular structure planning, when preparing spectrally bespoken materials for optoelectronic applications.

## Conclusion

To summarize, we have developed a new flexible synthetic approach to the preparation of highly luminescent donor–acceptor compounds, which are of potential interest for optoelectronics on the basis of the same precursors. These solution processable compounds consist of an azole-type central unit, symmetrically connected to alkyl (or alkoxy) bithiophenes. We demonstrate that the photoluminescence properties of this family of compounds can be tuned in a wide spectral range by changing the chemical nature of the central electron accepting ring and additionally by the type and the position of the solubilising substituent on the bithiophene unit.

## Supporting Information

File 1 Experimental part.
