# Supplementary material for: Synthesis of new, highly luminescent bis(2,2’-bithiophen-5-yl) substituted 1,3,4-oxadiazole, 1,3,4-thiadiazole and 1,2,4-triazole
Source: Beilstein J Org Chem. 2014 Jul 14;10:1596–602. doi: 10.3762/bjoc.10.165 (PMC4142979; doi:10.3762/bjoc.10.165)

Supporting Information

for

Synthesis of new, highly luminescent bis(2,2’-bithiophen-5-yl) substituted 1,3,4-oxadiazole, 1,3,4-thiadiazole and 1,2,4-triazole

Anastasia S. Kostyuchenko[1,2], Vyacheslav L.Yurpalov[1], Aleksandra Kurowska[3], Wojciech Domagala[3], Adam Pron[4] and Alexander S. Fisyuk*[1,2]

Address: [1]Department of Organic Chemistry, Omsk F. M. Dostoevsky State University, 55a Mira Ave, 644077 Omsk, Russia, [2]Laboratory of New Organic Materials, Omsk State Technical University, Mira Ave, 11, Omsk 644050, Russia, [3]Department of Physical Chemistry and Technology of Polymers, Silesian University of Technology, Marcina Strzody 9, 44-100 Gliwice, Poland and [4]Faculty of Chemistry Warsaw University of Technology, Noakowskiego 3, 00-664 Warszawa, Poland

* Corresponding author

Email: Alexander S. Fisyuk - fisyuk@chemomsu.ru

Experimental part

Table of Contents

General information..S2

Experimental procedures and analytical data...S3

References……………………………………………………………………….S11

General information

The IR spectra were recorded on an Infralum FT-801 spectrometer in KBr pellets for solids, or in thin films for liquid compounds. UV–vis spectra were taken in dichloromethane solutions using a Hewlett Packard 8453 diode-array spectrometer, while fluorescence spectra were recorded on Hitachi F-2500 fluorescence spectrometer. The frontier molecular orbital (HOMO–LUMO) gaps of investigated compounds were estimated from the onset of their π–π* absorption band. Fluorescence excitation spectra were recorded to determine the excitation wavelength at which the maximum fluorescence response is observed. Subsequent fluorescence emission spectra were recorded at excitation wavelengths determined in this manner. The quantum yield of examined compounds was determined relative to 9,10-diphenylanthracene – a known quantum yield standard, using comparative method [1,2]. Using this method, the quantum yield is calculated according to the following equation:

$$\Phi = \Phi_R \cdot \left(\frac{m}{m_R}\right) \cdot \left(\frac{n^2}{n_R^2}\right)$$

where: Φ is the quantum yield, *m* is the slope of origin approaching linear segment of the plot of integrated fluorescence intensity vs absorbance of a fluorophore solution, *n* is the refractive index of solvent and subscript *R* refers to data for the solution of reference fluorophore of known quantum yield – here: 9,10-diphenylanthracene in ethanol (Φ_R = 0.950 for excitation at 330–380 nm) and in cyclohexane (Φ_R = 0.955 for excitation at 366 nm) [3]. The ^{1}H and ^{13}C NMR spectra were obtained in $CDCl_3$ with TMS as an internal standard, using a Bruker DRX 400 spectrometer (400 and 100 MHz, respectively). The ^{13}C NMR spectra were obtained in the *J*-modulation mode. The elemental analyses were carried out on a Carlo Erba 1106 CHN analyzer. The melting points were determined on a Kofler bench. The reaction course and purity of the products were checked by thin-layer chromatography on Sorbfil UV-254 plates and were visualized

with UV light. All chemicals were of analytical grade and purchased from Sigma-Aldrich Chemical Co.

Experimental and analytical data

The synthesis of **1, 2, 3, 8, 10, 11, 15** is described in detail in *Supporting Information* of reference [4].

2,5-Bis(3-decyl-2,2'-bithiophene-5-yl)-1,3,4-oxadiazole (13). A mixture of **11** (0.41 g, 0.6 mmol) and phosphorus oxychloride (7.58 g, 49.4 mmol) was refluxed for 5 h (the completion of the reaction was monitored by TLC). The resulting solution was cooled to room temperature, and poured on a mixture of crushed ice (10 g) in water (50 mL). It was then neutralized by saturated solution of $NaHCO_3$. The product was extracted with $CHCl_3$ (3 × 20 mL). The extract was washed with saturated aqueous sodium chloride (30 mL), dried (Na_2SO_4) and concentrated. Then the solvent was evaporated under reduced pressure. The crude product was purified by column chromatography using benzene as eluent. Yield: 0.40 g (78%); yellow solid; R_f = 0.38 (silica gel, benzene or CH_2Cl_2) mp 86–87 °C

IR (KBr). ν cm^{-1}: 1580 (C=N).

^{1}H NMR (400 MHz, $CDCl_3$, δ, ppm, J, Hz): 0.88 (t, ^3J=6.7 Hz, 6 H, $2CH_3$), 1.23-1.40 (m, 28H, $14CH_2$), 1.69 (m, 2H, $ThCH_2C\underline{H}_2C_8H_{17}$), 2.79 (t, ^3J=7.8 Hz, 4H, 2Th-$C\underline{H}_2$), 7.10 (d.d, ^3J=4.9 Hz, ^3J=3.8 Hz, 2H, 2Th-4'-H), 7.23(d.d, ^3J=3.5 Hz, ^4J=0.5 Hz, 2H, 2Th-3'-H), 7.38 (d.d, ^3J=5.1 Hz, ^4J=0.6 Hz, 2H, 2Th-5'-H), 7.64(s, 2H, 2Th-4-H).

^{13}C NMR (100 MHz, δ, ppm): 14.12 (CH_3), 22.70, 29.27, 29.34, 29.43, 29.50, 29.58, 29.61, 30.47, 31.91 (CH_2), 121.99 (2-Th), 126.64 (3′-Th), 127.03(5′-Th), 127.70(4′-Th), 132.36 (2-Th), 134.76 (5-Th), 135.94 (3-Th), 140.51 (2′-Th), 160.01 (2,5-Oxadiazole)

Elemental analysis. Calculated for $C_{38}H_{50}N_2S_5$: %C = 65.66, %H = 7.25, %N = 4.03; found: %C = 65.56, %H = 7.28, %N = 4.13

Ethyl 3-oxo-3-(2-thienyl)propanoate (4). Compound **4** was prepared according to the known procedure [5] (see *Supporting Information*). Yield 87%. B.p. 164-168 °C (15-18 mmHg), lit. 143 °C (7 mmHg).

Ethyl 3-chloro-3-(2-thienyl)acrylate (5)

5.0 g (25 mmol) of freshly distilled compound **4** was slowly added with stirring to 11.5 mL (125 mmol) of phosphoryl chloride under cooling. Then 3.9 mL (28 mmol) of dry triethylamine was added drop-wise during ca. 30 min. After the addition of 0.1 mL of 1,8-diazabicyclo[5.4.0]undec-7-ene (DBU) the reaction mixture was stirred for 30 min in an ice bath, then for 6 h at 60–70 °C and finally left overnight at rt. The excess of $POCl_3$ was removed under vacuum and the resulting mixture was treated with 100 g of crushed ice. The product was extracted with dichloromethane (4 × 20 mL) and dried over sodium sulfate. After removal of the solvent on a rotary evaporator, the final residue was purified by column chromatography (SiO_2/hexane-ethyl acetate 9:1) yielding 4.1 g (76%) of yellow oil. R_f 0.35 (hexane-ethyl acetate 9:1).

IR (v, cm^{-1}): 1606 (C=C), 1721 (C=O), 2981 (C-H_{alk}), 3106 (C-H_{ar}).

NMR ^{1}H ($CDCl_3$, δ, ppm, J, Hz): 1.33 (3H, t, ^{3}J= 7.1, $OCH_2C\underline{H_3}$); 4.26 (2H, q, ^{3}J= 7.2, $OC\underline{H_2}CH_3$); 6.53 (1H, s, C(Cl)=C$\underline{H}$); 7.06 (1H, dd, ^3J=5.0, ^3J=3.8, Th-4-H); 7.41 (1H, dd, ^3J=5.1, ^4J=1.0, Th-5-H); 7.55 (1 H, dd, ^3J=3.8, ^4J=1.1, Th-3-H);

NMR ^{13}C ($CDCl_3$, δ, ppm): 14.27 ($OCH_2\underline{C}H_3$), 60.57($O\underline{C}H_2CH_3$), 113.39 (C(Cl)=$\underline{C}$H), 128.09 (3(C)- Th), 129.16 (5(C)- Th),129.46 (4(C)- Th), 141.09 (2(C)- Th), 164.01 (C=O).

Elemental analysis. Calculated for $C_9H_9ClO_2S$: %C = 49.89, %H = 4.19; found: %C = 48.56, %H = 4.28

Ethyl 4-hydroxy-2,2'-bithiophene-5-carboxylate (6). 2.2 mL (20 mmol) of ethyl thioglycolate was added to a solution of sodium ethylate, prepared from 0.92 g (40 mmol) of sodium and 75 mL of dry ethanol. The resulting mixture was stirred for an additional 15 min under nitrogen. Then the solution of 4.0 g (18 mmol) of **5** in 5 mL of dry ethanol was added dropwise to the resulting suspension and stirred for 1.5 h under an inert atmosphere. The reaction mixture was concentrated in vacuum, dissolved in 150 mL of water, cooled and acidified with 2 M HCl to pH 3–4. The precipitate was filtered out, then washed with water to pH 7. Recrystallization from methanol gave 2.2 g (49%) of white powder. Mp 70–71 °C (methanol).

IR (v, cm^{-1}): 1097 (C-O-C), 1645 (C=O), 3083 [Wight] (O-H).

NMR ^{1}H (CDCl$_3$, δ, ppm, J, Hz): 1.38 (3H, t, ^{3}J= 7.1, OCH$_2$C$\underline{H_3}$); 4.36 (2H, q, ^{3}J= 7.2, OC$\underline{H_2}$CH$_3$); 6.28 (1H, s, Th-3-H); 7.04 (1H, dd, ^{3}J=5.1, ^{3}J=3.7, Th-4'-H); 7.28 (1H, dd, ^{3}J=3.7, ^{4}J=1.2, Th-5'-H); 7.32 (1 H, dd, ^{3}J=5.1, ^{4}J=1.0, Th-3'-H), 9.69 (1H, s, OH);

NMR ^{13}C (CDCl$_3$, δ, ppm): 14.37 (OCH$_2$$\underline{C}H_3$), 61.07(O$\underline{C}H_2CH_3$), 114.96 (3'(C)- Th), 125.40 (3(C)- Th), 126.63 (5'(C)- Th), 128.14 (4'(C)- Th), 136.48 (4(C)- Th), 142.32 (2'(C)- Th), 164.47 (C=O).

Elemental analysis. Calculated for $C_{11}H_{10}O_3S_2$: %C = 51.95, %H = 3.96; found: %C = 52.06, %H = 4.03

Ethyl 4-(hexyloxy)-2,2'-bithiophene-5-carboxylate (7). Potassium *tert*-butylate (0.86 g, 7.7 mmol) was added to a solution of 1.7 g (6.7 mmol) of **6** in 14 mL of dry DMSO under nitrogen and the resulting mixture was stirred for 15 min. After the drop-wise addition of 1.56 g (7.2 mmol) of 1-iodohexane to this mixture, it was stirred under inert atmosphere for 4 h, then poured into 150 mL of ice water. The product was extracted with dichloromethane (4 × 25 mL). The organic layer was washed with brine, dried over $MgSO_4$ and concentrated in vacuum. Purification by the column chromatography

(SiO_2/hexane-ethyl acetate 9:1) yielded 1.83 g (81%) of **7** as a green solid. Mp 40–42 °C (hexane-ethyl acetate 9:1). R_f 0.39 (hexane-ethyl acetate 9:1).

IR (ν, cm^{-1}): 1092 (C-O-C), 1700 (C=O); 2851, 2953 (C-H_{alk}); 3098 (C-H_{ar}).

NMR ^{1}H ($CDCl_3$, δ, ppm, J, Hz): 0.92 (3H, t, ^3J= 7.0, $OCH_2C\underline{H_3}$); 1.33-1.38 (7H, m, $(C\underline{H}_2)_2$, CH_3) 1.50 (2H, m, CH_2), 1.83 (2H, dd, ^3J=14.9, ^3J=6.9, CH_2), 4.15 (2H, t, ^3J=6.7, $OC\underline{H}_2$), 4.31 (2H, q, ^3J=7.0, $OC\underline{H}_2CH_3$); 6.90 (1H, s, Th-3-H); 7.04 (1H, dd, ^3J=5.1, ^3J=3.5, Th-4'-H); 7.27 (1H, dd, ^3J=3.7, ^4J=1.2, Th-5'-H); 7.30 (1H, dd, ^3J=5.1, ^4J=1.2, Th-3'-H);

NMR ^{13}C ($CDCl_3$, δ, ppm): 14.00 ($OCH_2\underline{C}H_3$), 14.39 (CH_3), 22.55, 25.47, 29.30, 31.50 ($(CH_2)_4$), 60.42 ($O\underline{C}H_2CH_3$), 72.11 ($O\underline{C}H_2CH_2$), 112.94 (3(C)- Th), 124.97 (3'(C)- Th), 126.45 (5'(C)- Th), 128.12 (4'(C)- Th), 136.69 (2(C)- Th), 141.03 (2'(C)- Th), 161.09 (4(C)- Th), 161.70 (C=O).

Elemental analysis. Calculated for $C_{17}H_{22}O_3S_2$: %C = 60.32, %H = 6.55; found: %C = 60.38, %H = 6.61

4-(Hexyloxy)-2,2'-bithiophene-5-carboxylic acid (9). 1.0 g (3 mmol) of **7** dissolved in 5 mL of dry ethanol was added to a solution of 0.5 g (9 mmol) of potassium hydroxide in 8 mL of dry ethanol. The reaction mixture was heated to 50–60 °C and stirred for 4 h, then cooled to rt and concentrated in vacuum. The residue was dissolved in 20 mL of water and acidified with 2 M HCl to pH 3–4. The resulting suspension was vigorously stirred for 20 min and filtered out. The precipitate was washed with ice water and dried. Recrystallization from methanol gave 0.79 g (87%) of **9** as a white powder. Mp 134–136 °C (methanol).

IR (ν, cm^{-1}): 1097 (C-O-C), 1649 (C=O), 2556 (C(=O)$\underline{OH}$); 2857, 2947 (C-H_{alk}); 3088 (C-H_{ar}).

NMR ^{1}H ((CD$_3$)$_2$CO, δ, ppm, J, Hz): 0.83 (3H, t, ^3J= 7.0, CH$_2$C$\underline{H_3}$); 1.25-1.32 (4H, m, (C$\underline{H}_2$)$_2$) 1.45 (2H, m, CH$_2$), 1.75 (2H, m, CH$_2$), 2.68 (s, OH), 4.21 (2H, t, ^3J=6.7, OC$\underline{H}_2$); 7.06 (1H, dd, ^3J=5.1, ^3J=3.5, Th-4'-H); 7.19 (1H, s, Th-3-H); 7.38 (1H, dd, ^3J=3.7, ^4J=1.2, Th-5'-H); 7.47 (1H, dd, ^3J=5.1, ^4J=1.2, Th-3'-H);

NMR ^{13}C ((CD$_3$)$_2$CO, δ, ppm): 12.26 (CH$_3$), 21.24, 24.17, 28.13, 30.18 (4CH$_2$), 71.00 (O$\underline{C}$H$_2$CH$_2$), 107.37 (5(C)- Th), 112.59 (3(C)- Th), 124.34 (3'(C)- Th), 125.71 (5'(C)- Th), 127.27 (4'(C)- Th), 135.27 (2(C)- Th), 139.92 (2'(C)- Th), 159.68 (4(C)- Th), 160.23 (C=O).

Elemental analysis. Calculeted for $C_{15}H_{18}O_3S_2$: %C = 58.04, %H = 5.84; found: %C = 58.08, %H = 5.91

***N*,*N*'-Bis[4-(hexyloxy)-2,2'-bithiophen-5-oyl]hydrazine (12).** Oxalyl chloride (0.64 mL, 7.6 mmol) was added to a suspension of **9** (0.59 g, 1.9 mmol) in 6 mL of dry dichloromethane under cooling (ice bath). After the addition of one drop of DMF to the resulting mixture, it was stirred for 30 min under cooling then at rt for additional 2.5 h. The solvent and the excess of oxalyl chloride were removed under reduced pressure and the final product (acid chloride) was used in subsequent reactions without further purification. Crude acid chloride was dissolved in 12 mL of dichloromethane, cooled in an ice bath. Then, a mixture of 0.048 mL (0.95 mmol) of hydrazine hydrate and 0.92 mL (11 mmol) of dry pyridine in 3 mL of dichloromethane was added dropwise. The reaction mixture was stirred for 30 min under cooling and left overnight at rt. After removal of the solvent in vacuum the residue was treated with 10 mL of ice water and stirred for 15 min. The precipitate was filtered out, washed with water and dried. Recrystallization from methanol gave 0.35 g (60%) of **12** as a dark yellow solid. Mp 168–170 °C (methanol). R_f 0.67 (chloroform).

IR (ν, cm^{-1}): 1110 (C-O-C), 1608 (C=O); 2852, 2948 (C-H$_{alk}$); 3080 (C-H$_{ar}$), 3348 (N-H).

NMR ^{1}H ($CDCl_3$, δ, ppm, J, Hz): 0.91 (3H, m, $CH_2C\underline{H_3}$); 1.38 (4H, m, $(C\underline{H_2})_2$) 1.53 (2H, m, CH_2), 1.97 (2H, m, CH_2), 4.24 (2H, t, ^3J=6.7, $OC\underline{H_2}$); 6.92 (1H, s, Th-3-H); 7.05 (1H, m, Th-4'-H); 7.27 (1H, m, Thi-5'-H); 7.29 (1H, m, Th-3'-H), 10.13 (1H, s, NH);

NMR ^{13}C ($CDCl_3$, δ, m.d.): 14.02 ($CH_2\underline{C}H_3$), 22.59, 25.41, 29.19, 31.39 ($(CH_2)_4$), 72.70 ($O\underline{C}H_2CH_2$), 111.91 (5(C)- Th), 112.01 (3(C)- Th), 125.01 (3'(C)- Th), 126.12 (4'(C)- Th), 128.21 (5'(C)- Th), 136.71 (2(C)- Th), 140.88 (2'(C)- Th), 156.50 (4(C)- Th), 156.75 (C=O).

Elemental analysis. Calculeted for $C_{30}H_{36}N_2O_4S_4$: %C = 58.41, %H = 5.88, %N = 4.54; found: %C = 58.43, %H = 5.90, N = 4.60

2,5-Bis[4-(hexyloxy)-2,2'-bithiophen-5-yl]-1,3,4-oxadiazole (14). A mixture of 0.20 g (0.32 mmol) of **12** and 3 mL of phosphoryl chloride was heated to 60–70 °C and stirred for 6 h, then cooled to rt, poured in small portions into 100 g of crushed ice. The product was extracted with chloroform (4 × 20 mL). The combined organic layers were washed with 30 mL of ice water and the solvent was removed in vacuum. Chromatography purification (SiO_2/benzene-ethyl acetate 20:1) of the final residue gave 0.14 g (83%) of **14** as a bright yellow powder. Mp 104–106 °C (chloroform). R_f 0,53 (chloroform).

IR (ν, cm^{-1}): 1113 (C-O-C); 1572, 1593 (C=N); 2852, 2926 ($C\text{-}H_{alk}$); 3079, 3109 ($C\text{-}H_{ar}$).

NMR ^{1}H ($CDCl_3$, δ, ppm, J, Hz): 0.89 (6H, m, $CH_2C\underline{H_3}$); 1.33 (8H, m, $(C\underline{H_2})_2$) 1.55 (4H, m, CH_2), 1.89 (4H, m, CH_2), 4.22 (4H, t, ^3J=6.5, $OC\underline{H_2}$); 6.96 (2H, s, Th-4-H); 7.05 (2H, dd, ^3J=5.1, ^4J=3.5, Th-4'-H); 7.27 (2H, dd, ^3J=3.6, ^4J=1.1, Thi-5'-H); 7.30 (2H, dd, ^3J=5.2, ^4J=1.1, Th-3'-H);

NMR ^{13}C ($CDCl_3$, δ, m.d.): 14.06 ($CH_2\underline{C}H_3$), 22.63, 25.59, 29.47, 31.65 ($(CH_2)_4$), 72.38 ($O\underline{C}H_2CH_2$), 101.38 (2(C)- Th), 113.24 (4(C)- Th), 124.79 (3'(C)- Th), 125.96 (5'(C)- Th), 128.17 (4'(C)- Th), 136.57 (5(C)- Th), 139.28 (2'(C)- Th), 158.06 (C=N), 158.64(4(C)- Th).

Elemental analysis: Calculeted for $C_{30}H_{34}N_2O_3S_4$: %C = 60.17, %H = 5.72, %N = 4.68; found: %C = 60.20, %H = 5.78, N = 4.70

3-Decyl-*N*-phenyl-2,2'-bithiophene-5-carboxamide (16). Oxalyl chloride (2.9 mL, 34 mmol) was added drop-wise into an anhydrous CH_2Cl_2 solution of 3-decyl-2,2'-bithiophene-5-carboxylic acid (**8**, 1.99 g, 5.7 mmol) under vigorous stirring at 0 °C. The reaction mixture was then additionally stirred at room temperature overnight. The solvent and the excess of oxalyl chloride were evaporated under reduced pressure. The resulting 3-decyl-2,2'-bithiophene-5-carboxylic acid chloride was used without purification in the subsequent reaction. Aniline (5.7 mmol) was dissolved in dry ethyl acetate (10 mL) in the presence of triethylamine (1.1 mL, 7.9 mmol). A solution of the acid chloride (5.7 mmol) in 10 mL dry ethyl acetate was added drop-wise. A precipitate was formed, and the mixture was stirred at room temperature overnight. Then, the solvent was removed under reduced pressure. Water (7 mL) was added, and the solid was collected on a filter, then washed three times with water. The resulting crude product was purified by crystallization from methanol.

Yield: 2.06 g (82%); white solid; R_f = 0.59 (silica gel, hexane/EtOAc, 5/1) mp 88–90 °C (methanol)

IR (v, cm^{-1}): 1631 (C=O), 3340 (N-H).

^{1}H NMR (400 MHz, $CDCl_3$, δ, ppm, J, Hz): 0.88 (t, ^3J=6.8 Hz, 3 H, CH_3), 1.25-1.35 (m, 14H, 7CH_2), 1.62 (m, 2H, $ThCH_2C\underline{H}_2C_8H_{17}$), 2.72 (t, ^3J=7.8 Hz, 4H, Th-$C\underline{H}_2$), 7.06-7.08 (d.d, ^3J=5.1 Hz, ^3J=3.6 Hz, 2H, 2Th-4'-H), 7.10-7.14 (m, 1H, Ph-4-H), 7.17 (d.d, ^3J=3.6 Hz, ^4J=1.2 Hz, 2H, 2Th-3'-H), 7.31-7.35 (m, 2H, Ph-3,5-H), 7.35 (d.d, ^3J=5.2 Hz, ^3J=3.6 Hz, H, Th-4'-H), 7.36(d.d, ^3J=5.1 Hz, ^4J=1.2 Hz, 2H, 2Th-5'-H), 7.49 (s, 1H, Th-4-H), 7.61-7.63 (m, 2H, Ph-3,5), 7.88 (s, 1H, -N$\underline{H}$-Ph).

^{13}C NMR (100 MHz, δ, ppm): 14.11 (CH_3), 22.69, 29.33, 29.42, 29.48, 29.58, 29.61, 30.53, 31.91 (CH_2), 120.30 (2,6-Ph), 124.55 (4-Ph), 126.51(3′-Th), 126.99 (4′-Th), 127.62 (5′-Th), 129.07 (3,5-Ph), 131.52 (4-Th), 135.05 (2-Th), 135.80 (3-Th), 136.29 (5-Th), 137,73 (2′-Th), 140.25 (1-Ph) 159.92 (-C(O)-NHPh).

Elemental analysis: Calculeted for $C_{25}H_{31}NOS_2$: %C =70.54**,** %H =7.34, %N =3.29; found: %C = 70.46, %H = 7.40, %N = 3.33

3,5-Bis(3-decyl-2,2'-bithiophen-5-yl)-4-phenyl-4*H*-1,2,4-triazole (18). PCl_5 (0.23 g, 1.13 mmol) was added to a solution of 3-decyl-*N*-phenyl-2,2'-bithiophene-5-carboxamide (**16**, 0.50 g, 1.13 mmol) in 3 mL of anhydrous benzene placed in an ice bath. Then, the reaction mixture was stirred for 12 h at rt. After removing the solvent under reduced pressure, the crude product was washed with dry methanol by decantation. Residual amounts of methanol were removed on a vacuum evaporator. 3-Decyl-*N*-phenyl-2,2'-bithiophene-5-carboximidoyl chloride (**17**) was used at once in the subsequent reaction.

A mixture of **17** (1.13 mmol) and hydrazide **10** (0.41 g, 1.13 mmol) in anhydrous *N*,*N*-dimethylacetamide (5 mL) was heated at 165 °C for 6 h under nitrogen (the completion of the reaction was monitored by TLC). The solvent was removed under reduced pressure. **17** was purified by recrystallization from hexane or by column chromatography using benzene/EtOAc (20/1) or CH_2Cl_2 as eluent.

Yield: 0.52g (62%); white solid; R_f = 0.29 (silica gel, /EtOAc, 20/1) mp 96–98 °C

IR (ν, cm^{-1}) : 1497 (N=N)

^{1}H NMR (400 MHz, $CDCl_3$, δ, ppm, J, Hz): 0.89 (t, ^{3}J=6.8 Hz, 6 H, 2CH_3), 1.23-1.32 (m, 28H, 14CH_2), 1.45 (m, 2H, ThCH_2C̲H̲$_2C_8H_{17}$), 2.59(t, ^{3}J=7.7 Hz, 4H, 2Th-C̲H̲$_2$), 6.83 (s,2Th-4-H), 7.02 (d.d, ^{3}J=5.1 Hz, ^{3}J=3.5 Hz, 2H, 2Th-4'-H), 7.06 (d.d, ^{3}J=3.5 Hz,

^{4}J=1.2 Hz, 2H, 2Th-3'-H), 7.29 (d.d, ^{3}J=5.1 Hz, ^{4}J=1.2 Hz, 2H, 2Th-5'-H), 7.45-7.48 (m, 2H, Ph-2,6-H), 7.61-7.71 (m, 3H, Ph-3,5,4-H).

^{13}C NMR (100 MHz, δ, ppm): 14.12 (CH_3), 22.70, 28.96, 29.27, 29.35, 29.40, 29.54, 29.62, 30.18, 31.93 (CH_2), 125.39 (2-Th), 125.99 (2,6-Ph), 126.52 (4-Ph), 127.46 (3′-Th), 128.97 (5′-Th), 130.42 (4′-Th), 130.58 (3,5-Ph), 131.11 (4-Th), 133.58 (5-Th), 134.45 (3-Th), 135.09 (2′-Th), 139.61 (1,3,4-Triazole), 150.23 (1-Ph).

Elemental analysis: Calculated for $C_{44}H_{55}N_3S_4$: %C =70.07**,** %H =7.35, %N =5.57; found: %C = 70.06, %H = 7.38, %N = 5.60

References

1. Williams, A. T. R.; Winfield, S. A.; Miller, J. N. *Analyst*, **1983**, *108*, 1067-1071.

2. Allen, M. W.; Measurement of Fluorescence Quantum Yields, Thermo Fisher Scientific technical note 52019, Madison, WI, USA.

3. Brouwer, A. M. *Pure and Applied Chemistry*, **2011**, *83*, 2213-2228.

4. K. Kotwica, E. Kurach, G. Louarn, A. S. Kostyuchenko, A. S. Fisyuk, M. Zagorska, A. Pron. *Electrochimica Acta,* **2013**, *111*, 491– 498

5. Y. Jiang; X. Chen; Y. Zheng; Z. Xue; C. Shu; W. Yuan; X. Zhang, *Angew. Chem. Int. Ed*., **2011**, *50*, 7304–7307

2,5-bis(3-decyl-2,2'-bithiophene-5-yl)-1,3,4-oxadiazole (BThOxDiaz) (13)

^1H NMR (400 MHz, $CDCl_3$)

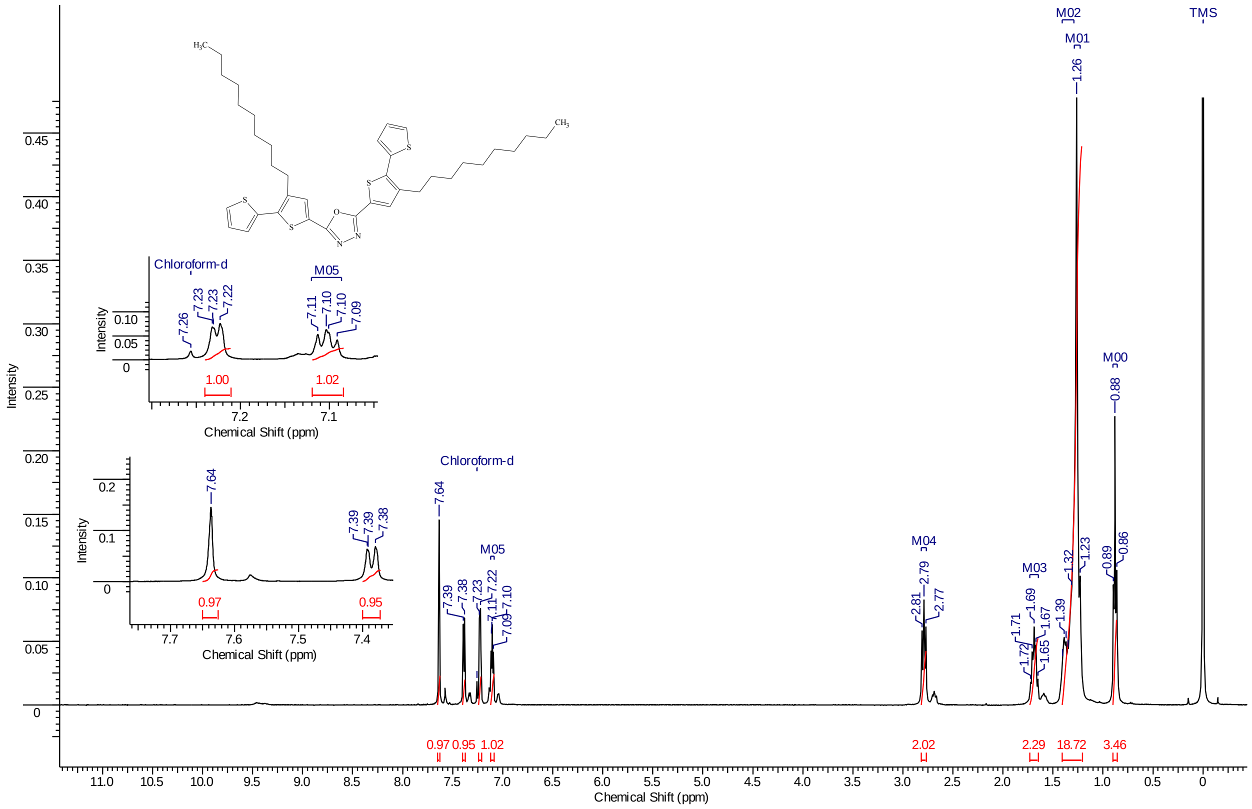

^{13}C NMR (100MHz)

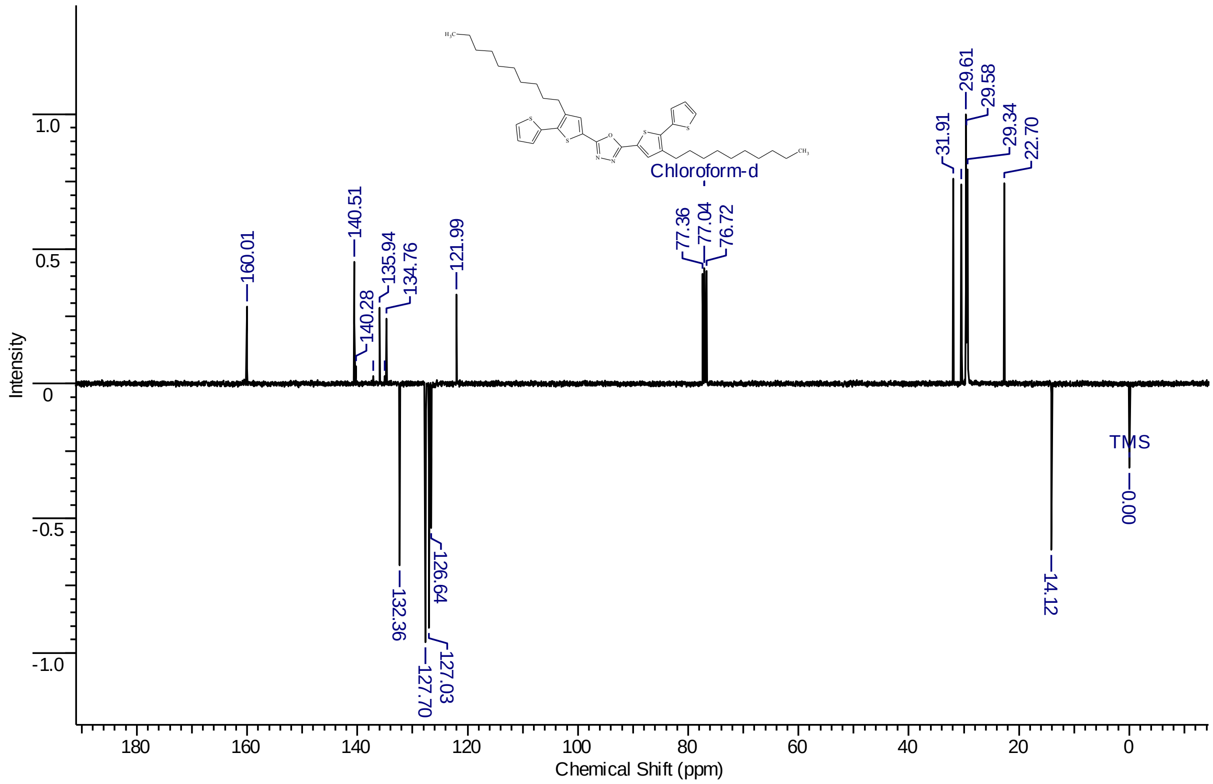

Ethyl 3-chloro-3-(2-thienyl)acrylate (5)

^1H NMR (400 MHz, $CDCl_3$)

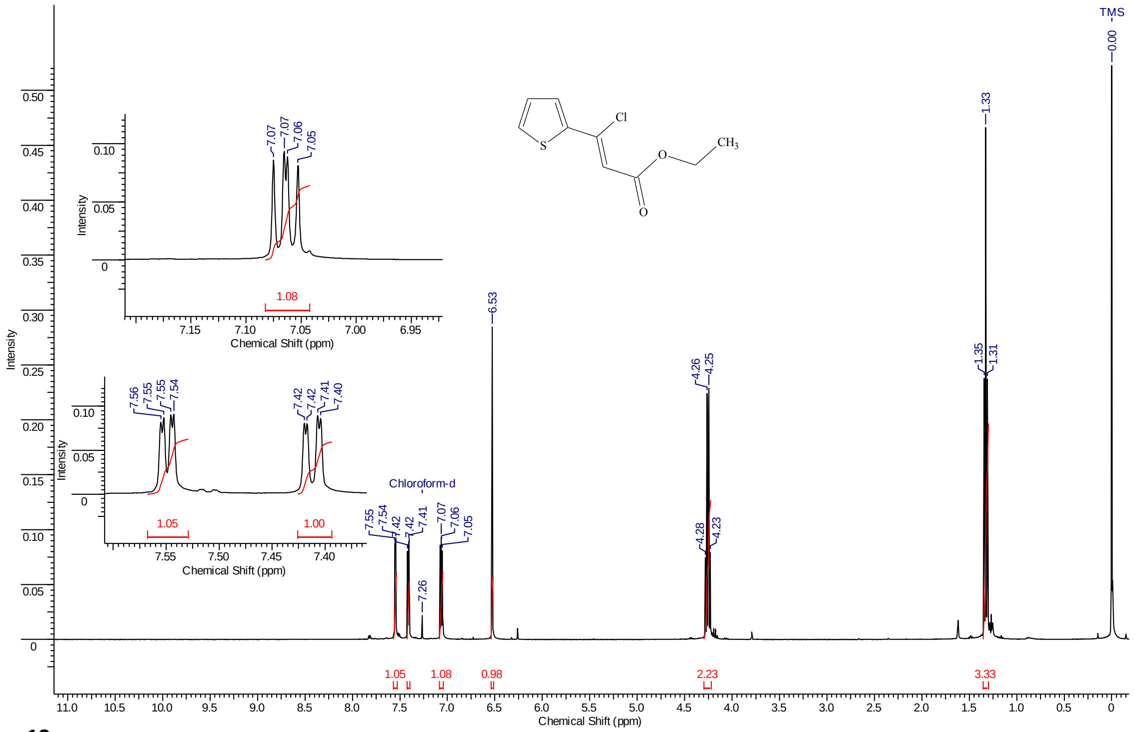

^{13}C NMR (100MHz)

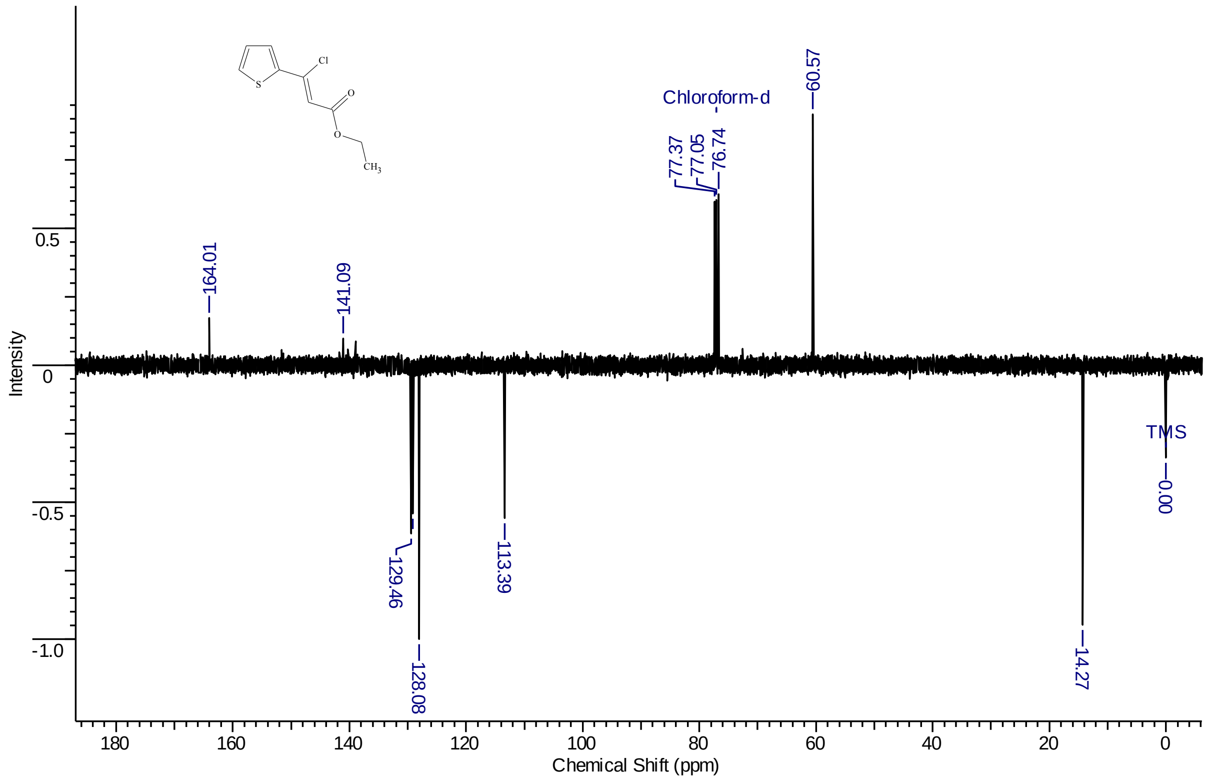

Ethyl 4-hydroxy-2,2'-bithiophene-5-carboxylate (6)

^{1}H NMR (400 MHz, $CDCl_3$)

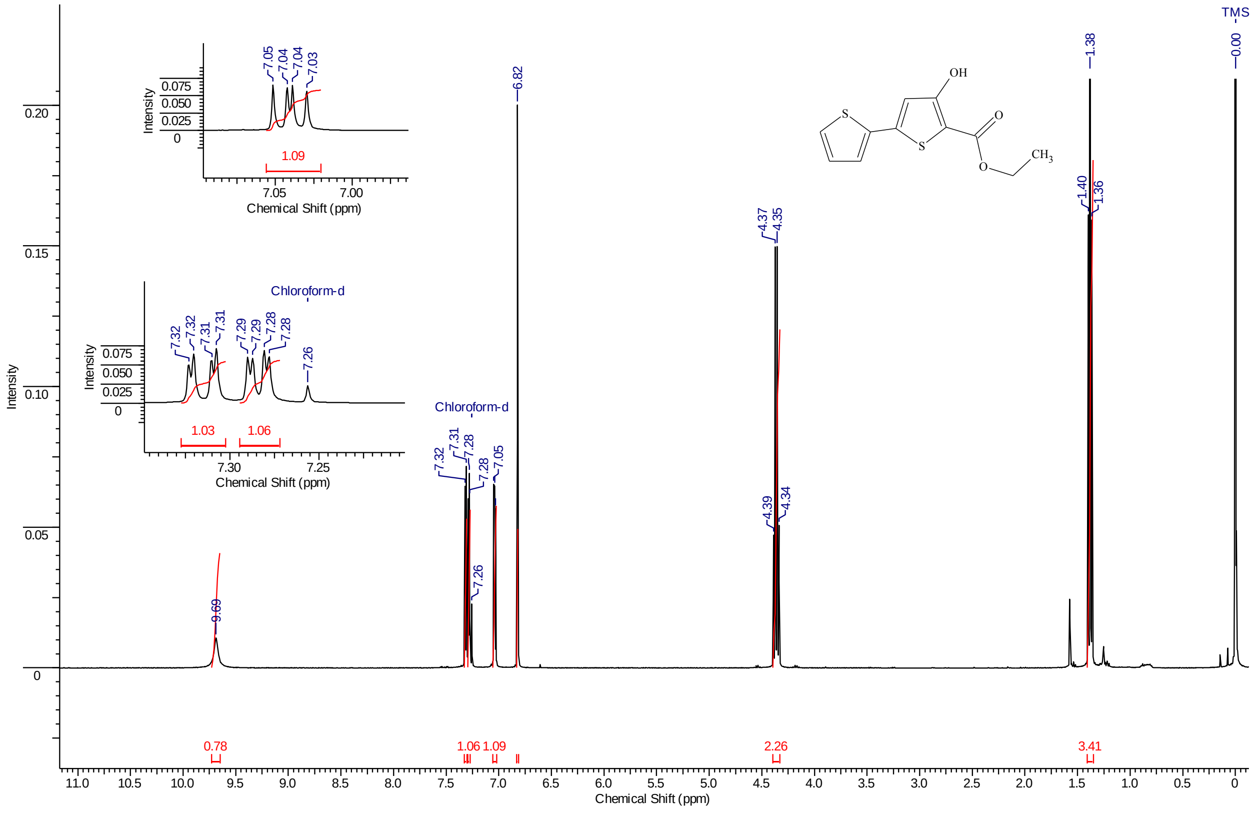

^{13}C NMR (100MHz)

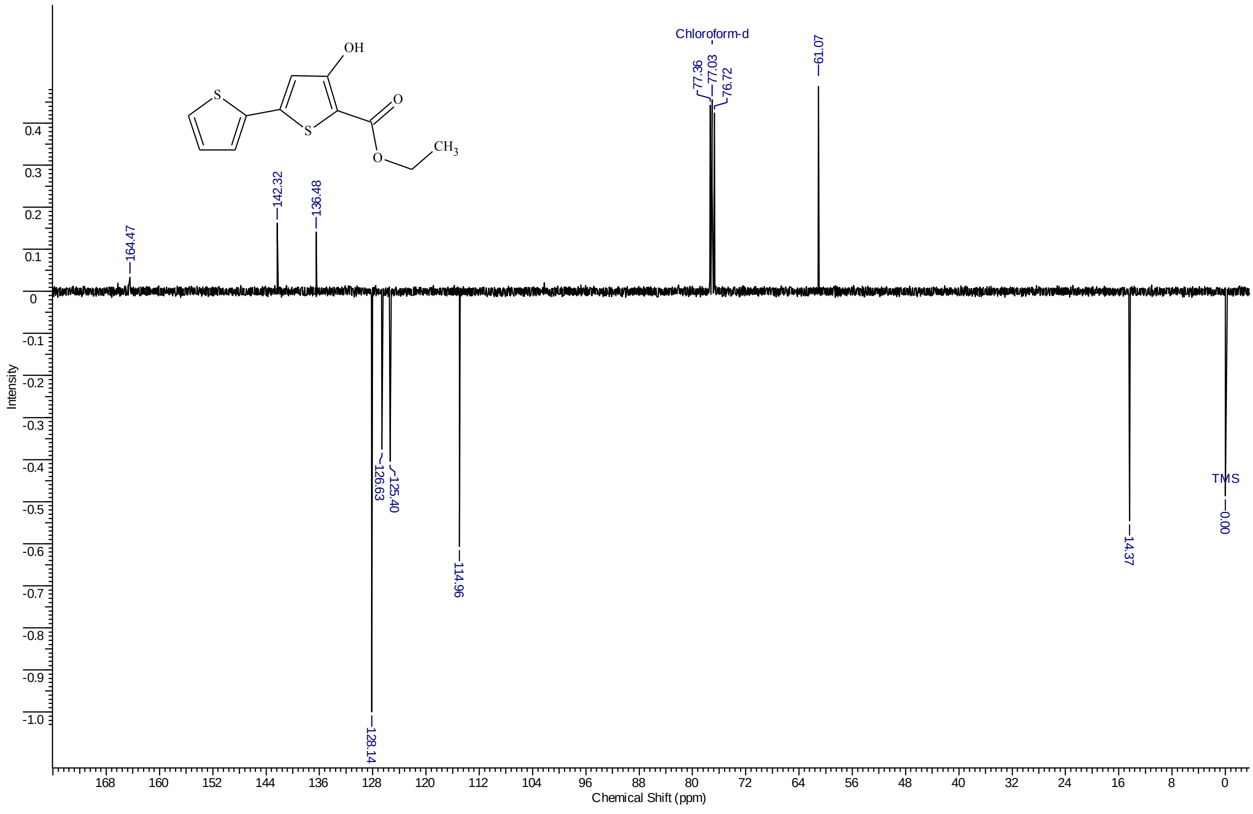

Ethyl 4-(hexyloxy)-2,2'-bithiophene-5-carboxylate (7).

^{1}H NMR (400 MHz, $CDCl_3$)

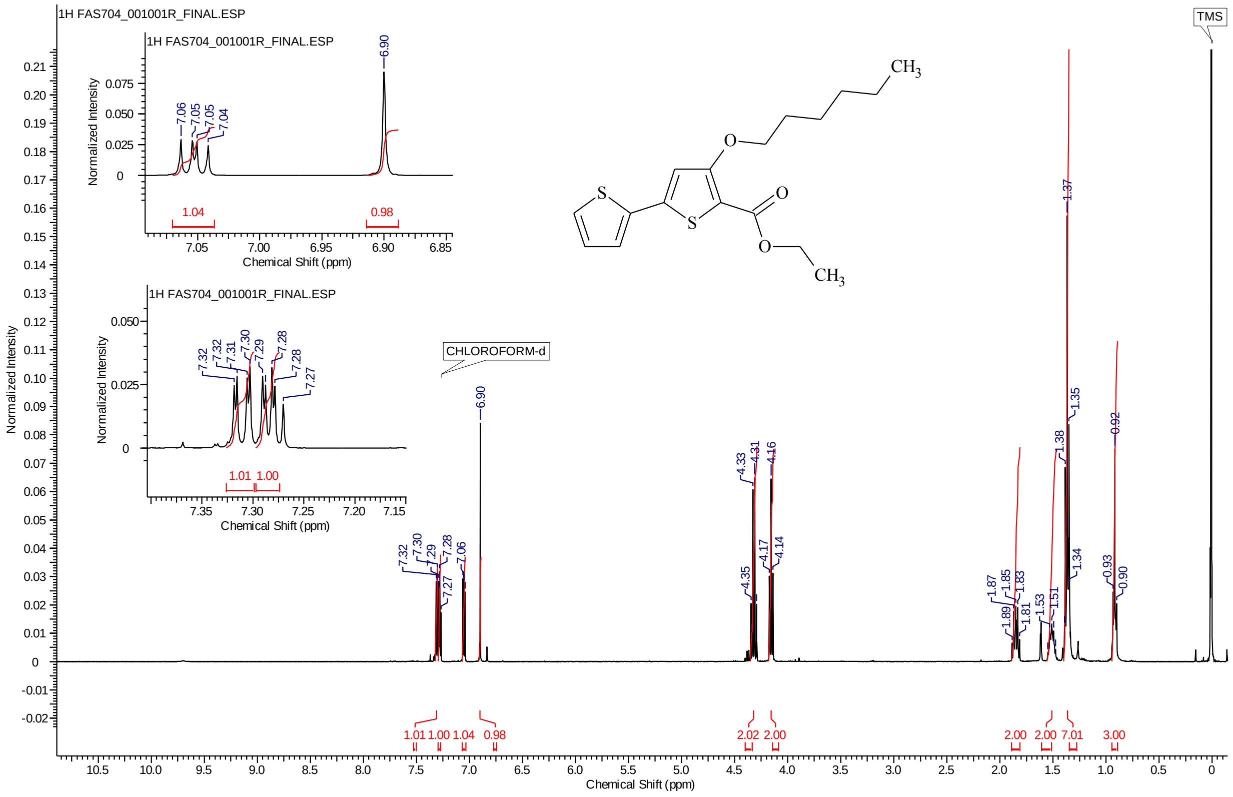

^{13}C NMR (100MHz)

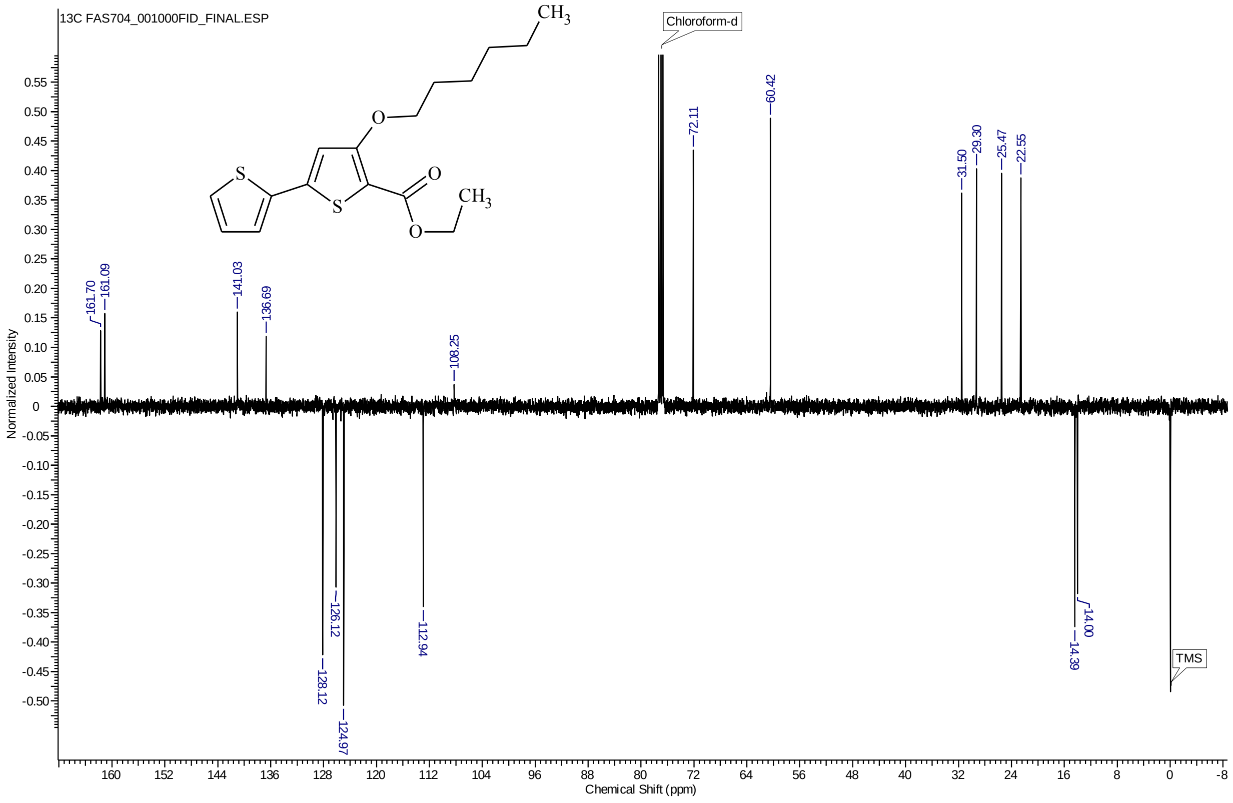

4-(Hexyloxy)-2,2'-bithiophene-5-carboxylic acid (9).

^{1}H NMR (400 MHz, $(CD_3)_2CO$)

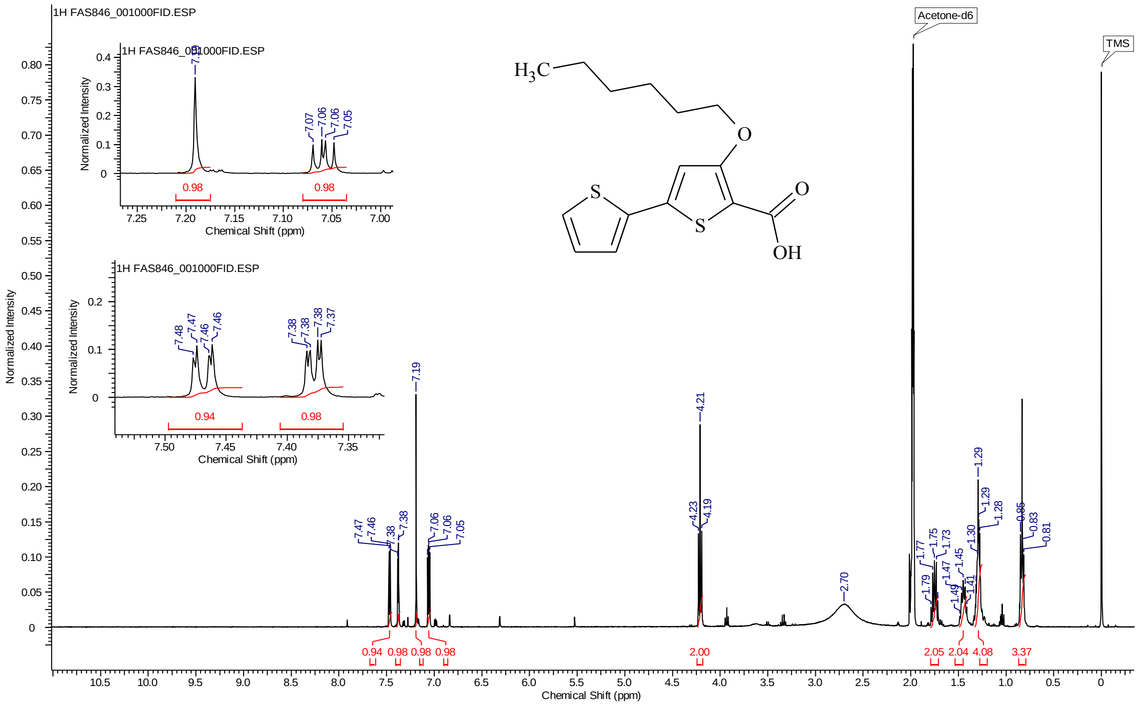

^{13}C NMR (100MHz)

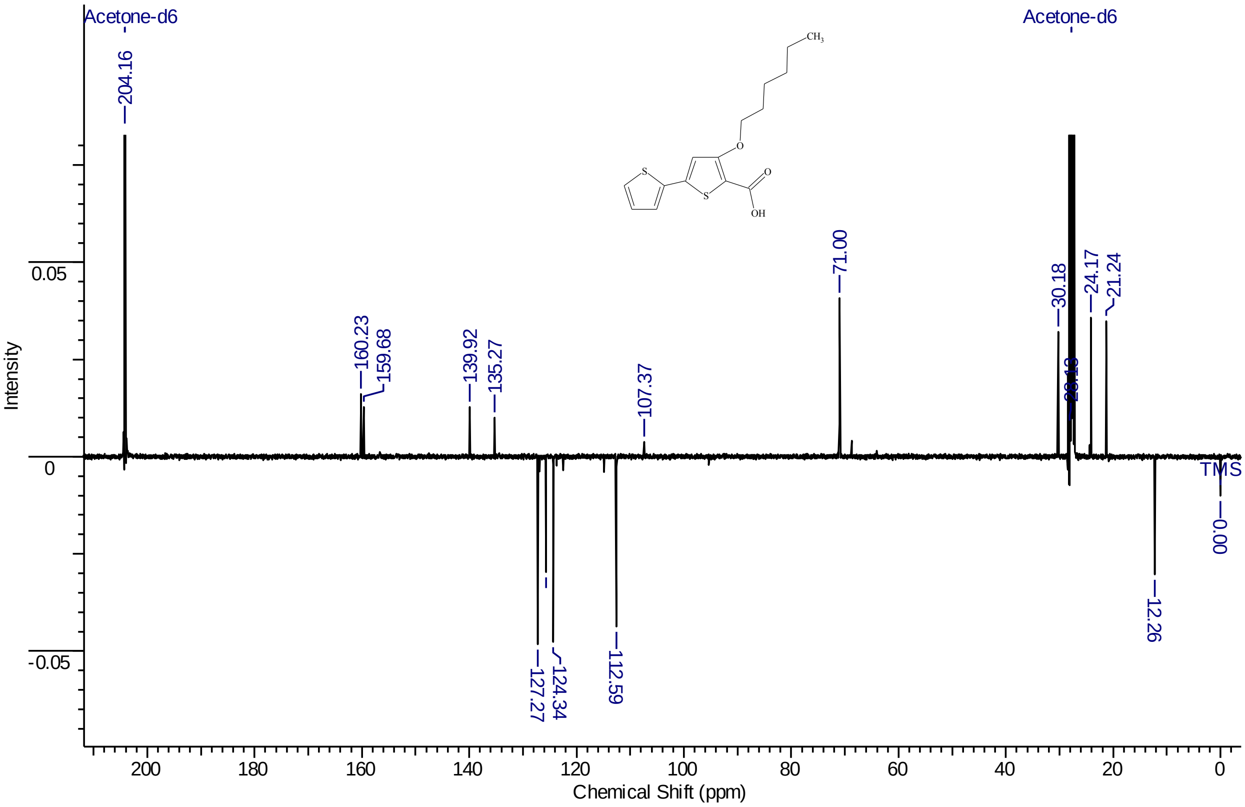

N,*N*'-Bis[4-(hexyloxy)-2,2'-bithiophen-5-oyl]hydrazine (12)

^{1}H NMR (400 MHz, $CDCl_3$)

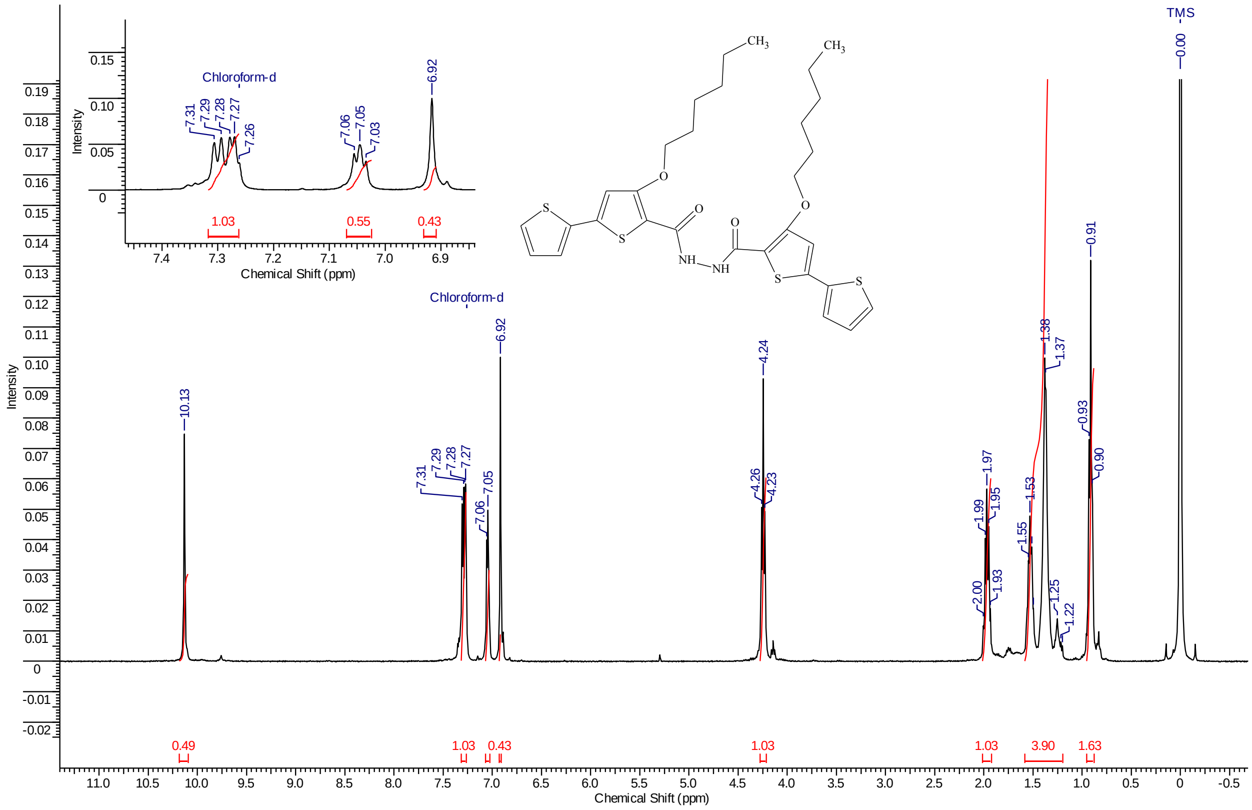

^{13}C NMR (100MHz)

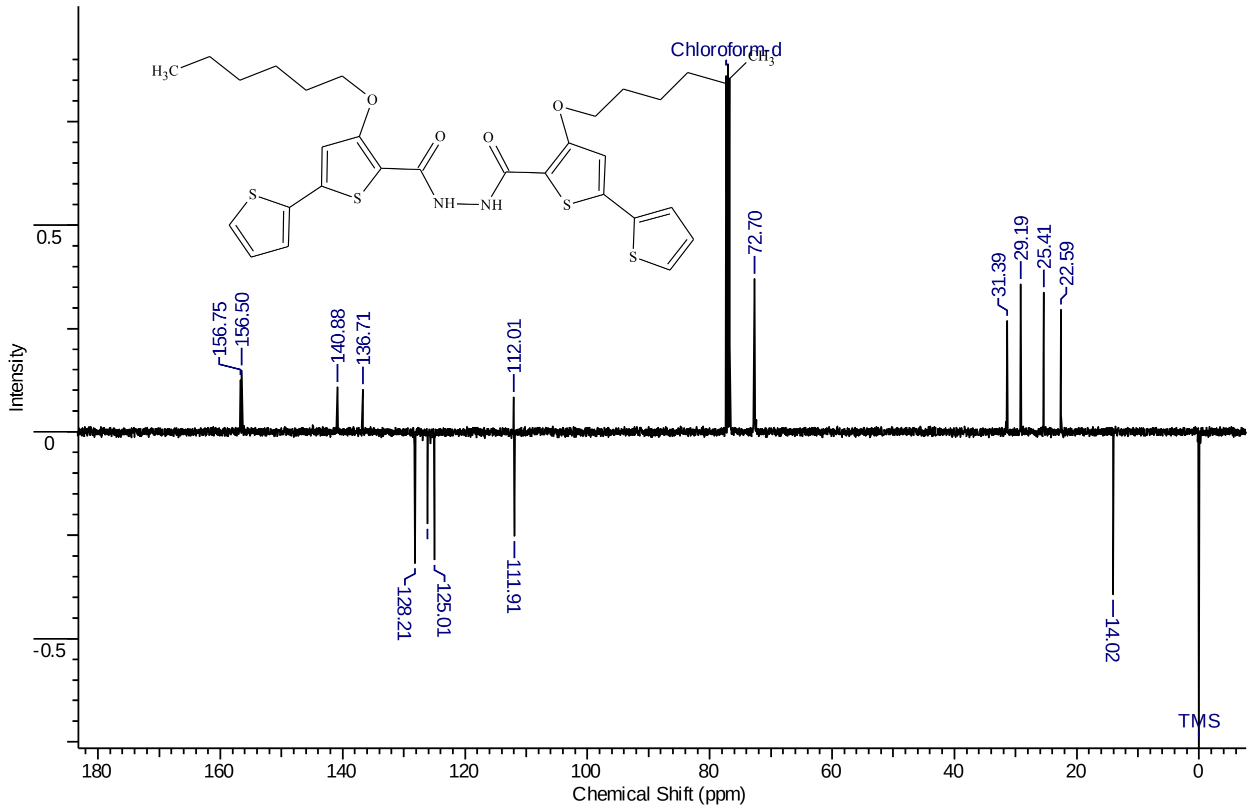

2,5-Bis[4-(hexyloxy)-2,2'-bithien-5-yl]-1,3,4-oxadiazole (14)

^{1}H NMR (400 MHz, $CDCl_3$)

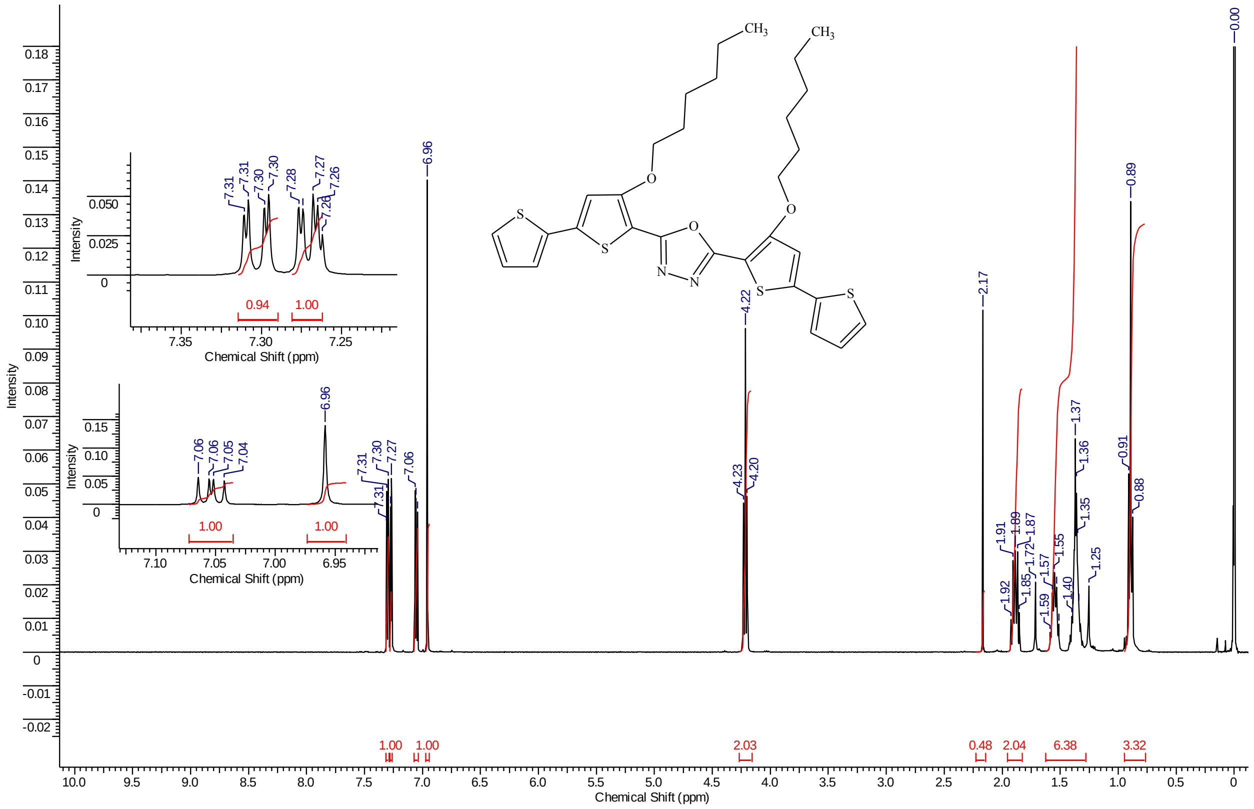

^{13}C NMR (100MHz)

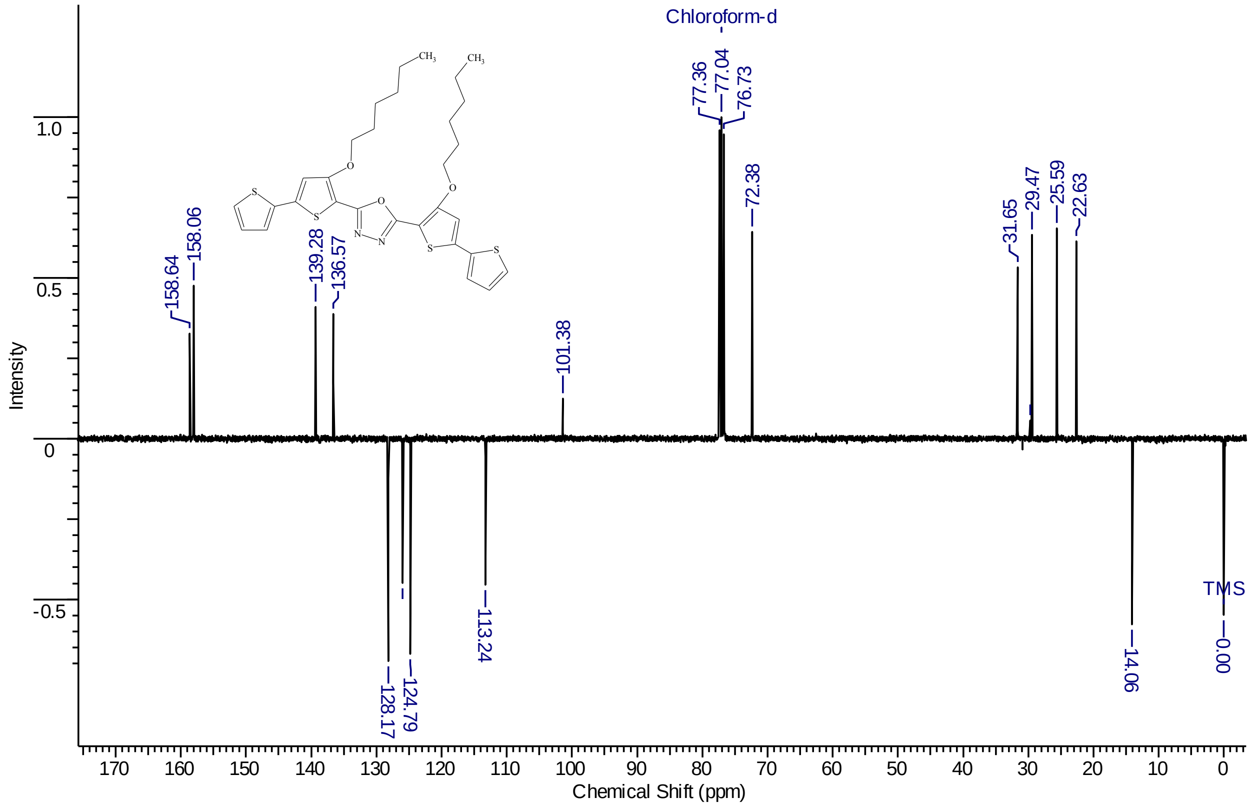

3-Decyl-*N*-phenyl-2,2'-bithiophene-5-carboxamide (16)

^{1}H NMR (400 MHz, $CDCl_3$)

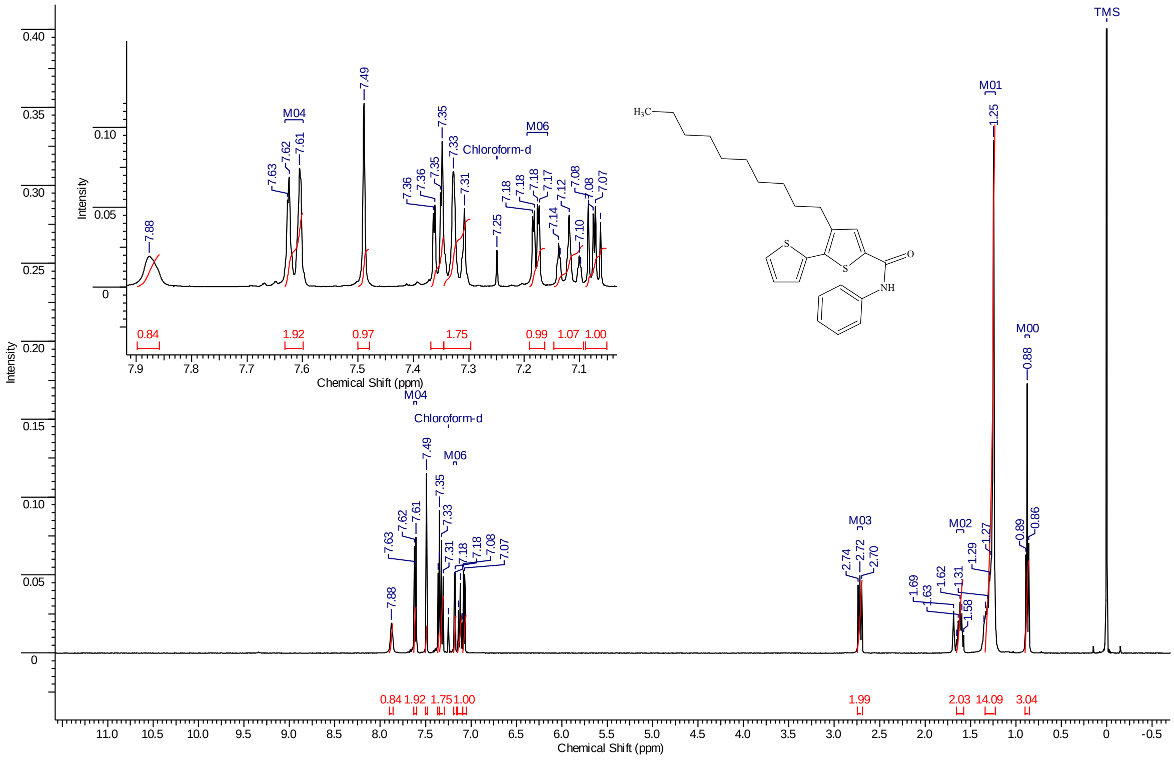

^{13}C NMR (100MHz)

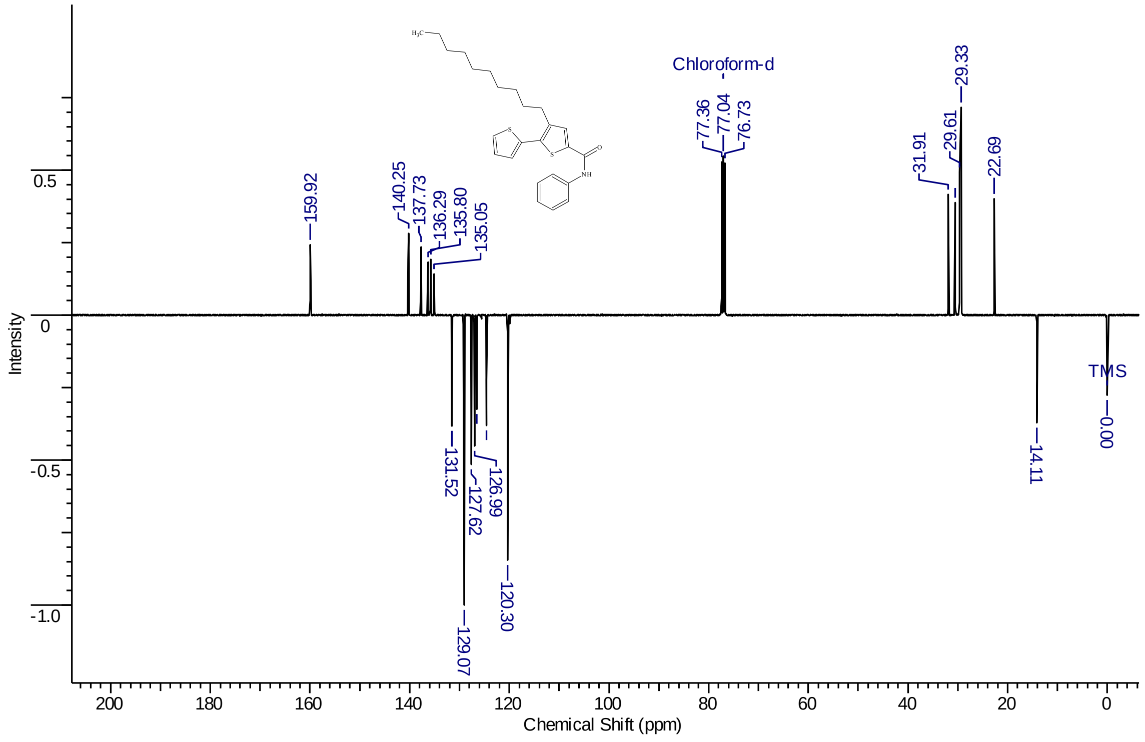

3,5-Bis(3-decyl-2,2'-bithiophen-5-yl)-4-phenyl-4*H*-1,2,4-triazole (BThPhTriaz) (18)

^{1}H NMR (400 MHz, $CDCl_3$)

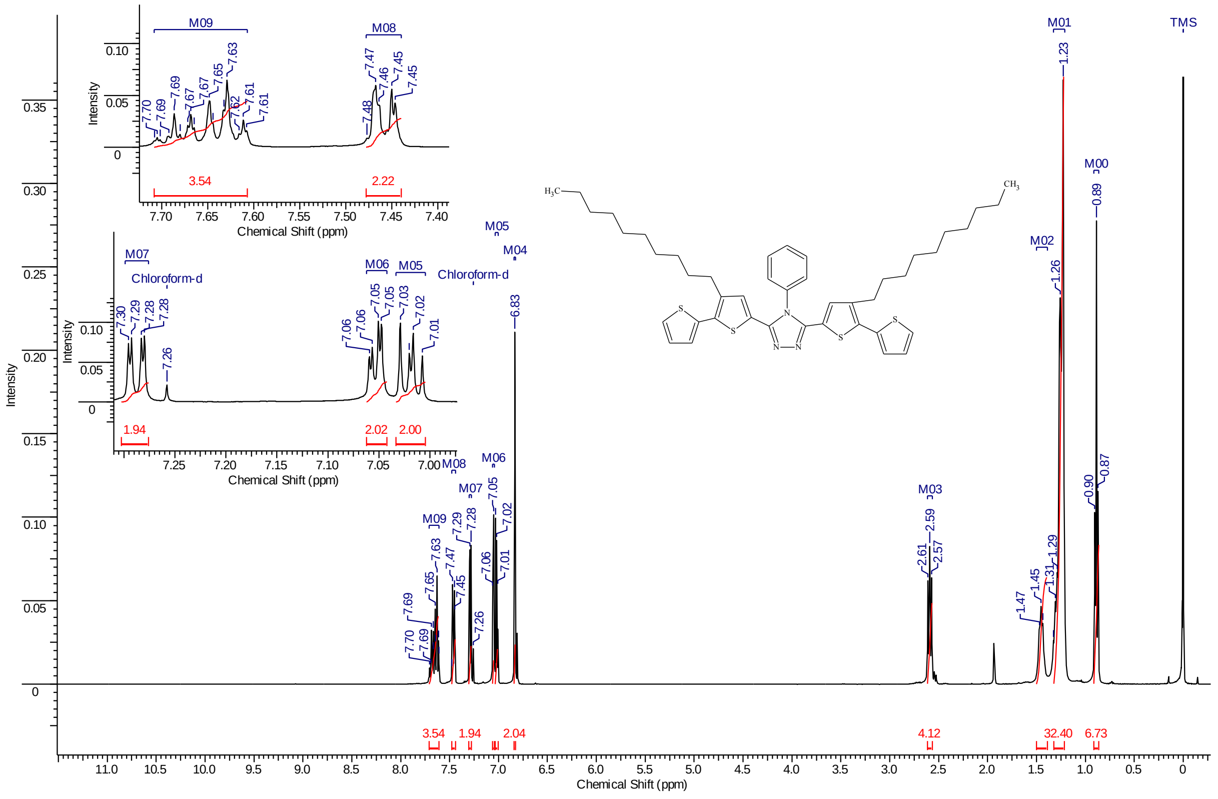

^{13}C NMR (100MHz)

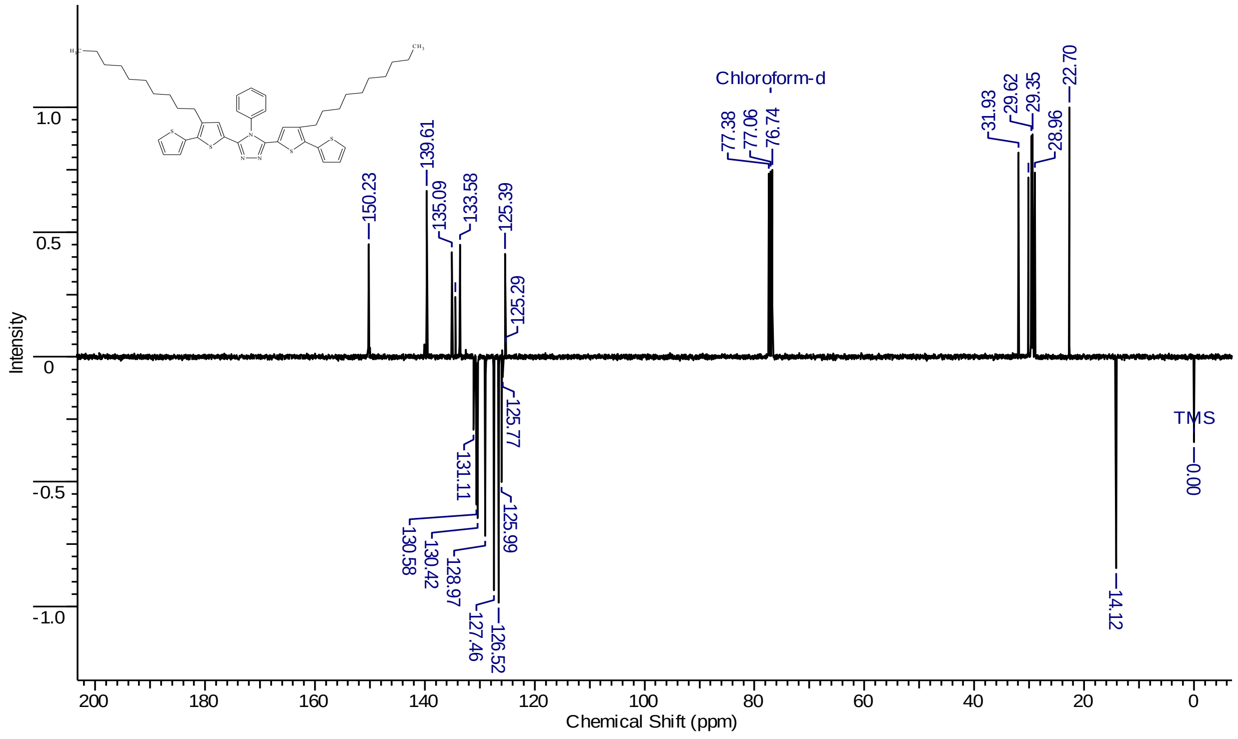

Supplement: File 1 — Experimental part. [file Beilstein_J_Org_Chem-10-1596-s001.pdf]
